# Job Strain and Late-Life Cognition: Findings From the Puerto Rican Elderly Health Conditions Study

**DOI:** 10.1093/geroni/igaa057.1173

**Published:** 2020-12-16

**Authors:** Taylor Vigoureux, Monica Nelson, Ross Andel, Brent Small, Michael Crowe, Ana Luisa Davila, Taylor Drury

**Affiliations:** 1 University of South Florida, Tampa, Florida, United States; 2 University of Alabama at Birmingham, Birmingham, Alabama, United States; 3 University of Puerto Rico, San Juan, Puerto Rico

## Abstract

Chronic stress at work, represented by job strain, has been associated with adverse late-life cognitive outcomes in the United States and Western Europe. We examined the relationship between job strain and change in cognition in a less affluent, Hispanic sample of adults aged 60-100 from mainland Puerto Rico. Job strain indicators (i.e., job demands/job control/job strain) were quantified from (a) standardized occupation-based job strain scores from Karasek’s Job Content Questionnaire (JCQ; n=1102), and (b) O*Net variables forming factors of job demands and job control (n=1639). Occupation information, covariates, and cognition came from the Puerto Rican Elderly Health Conditions (PREHCO) Study conducted in 2002-2003, with cognition follow-up in 2006-2007. All analyses controlled for age, sex, baseline depressive symptoms, baseline financial problems, and childhood economic hardship. Across both operationalizations of job strain indicators, higher job control was associated with less decline in late-life cognition (JCQ: b=.18, p<.05; O*Net: b=.31, p<.001) until controlling for education (JCQ: b=.09, p=.248; O*Net: b=.12, p=.097). Job strain was associated with more decline in cognition (JCQ: b=-.75, p<.05; O*Net: b=-.87, p<.05) until controlling for education (JCQ: b=-.49, p=.098; O*Net: b=-.46, p=.262). For Karasek’s measure, the relationships were driven by more educated participants. Job control was related to less cognitive decline whereas strain related to more decline among older Puerto Ricans over four years, whether assessed with JCQ-based or O*Net-based scores. However, education emerged as more important for change in late-life cognition than job strain indicators overall, suggesting results that diverge from countries with higher average socioeconomic status.

